# Sol-Gel Synthesis of Ordered β-Cyclodextrin-Containing Silicas

**DOI:** 10.1186/s11671-016-1380-2

**Published:** 2016-03-31

**Authors:** Iryna Mykolaivna Trofymchuk, Nadiia Roik, Lyudmila Belyakova

**Affiliations:** Chuiko Institute of Surface Chemistry, NAS of Ukraine, 17 General Naumov Str, Kyiv, 03164 Ukraine

**Keywords:** MCM-41, β-Cyclodextrin, Sol-gel synthesis, Postsynthesis grafting, Organosilane, Activating agent, 81.07.-b, 81.07.Pr, 81.20.Fw, 82.80.Ej, 82.80.Gk

## Abstract

New approaches for β-cyclodextrin-containing silicas synthesis were demonstrated. Materials with hexagonally ordered mesoporous structure were prepared by postsynthesis grafting and by co-condensation methods. β-Cyclodextrin activated by a *N*,*N*′-carbonyldiimidazole was employed for postsynthesis treatment of 3-aminopropyl-modified MCM-41 support as well as for sol-gel synthesis with β-cyclodextrin-containing organosilane and tetraethyl orthosilicate participation in the presence of cetyltrimethylammonium bromide. The successful incorporation of cyclic oligosaccharide moieties in silica surface layer was verified by means of FT-IR spectroscopy and chemical analysis. Obtained β-cyclodextrin-containing materials were characterized by X-ray diffraction, transmission electron microscopy, and low-temperature adsorption-desorption of nitrogen. In spite of commensurable loading of β-cyclodextrin groups attained by both proposed approaches (up to 0.028 μmol · m^–2^), it was found that co-condensation procedure provides uniform distribution of β-cyclodextrin functionalities in silica framework, whereas postsynthesis grafting results in modification of external surface of silica surface. Adsorption of benzene from aqueous solutions onto the surface of β-cyclodextrin-containing materials prepared by co-condensation method was studied as the function of time and equilibrium concentration. Langmuir and Freundlich models were used to evaluate adsorption processes and parameters. Adsorption experiments showed that β-cyclodextrin-containing silicas could be promising for the trace amount removal of aromatics from water.

## Background

M41S are ordered mesoporous materials with well-defined uniform pores, high surface area and pore volume, which have attracted great interest since 1992. MCM-41, one of the most widely studied M41S materials, consists of an amorphous (alumina, metallo) silicate framework forming hexagonal pores with diameter more than 1.5 nm [1]. The high potential applications of MCM-41 materials as adsorbents [2–4], catalysts [5–7], membranes [8, 9], and drug delivery systems [10–18] have been possible by means of their functionalization with organic compounds. Here, mesoporous silicas are the common basement for obtaining products with unique characteristics. The presence of highly reactive silanol groups in sufficiently large and tunable uniform pores opens up the possibility for introduction of various organic functional moieties, such as methyl and/or trimethylsil [11, 19], chloropropyl [20], aminoalkyl and triaminoalkyl [7, 9, 12, 21, 22], phenyl [23], mercaptoalkyl [24, 25], and sulfo [25, 26] groups into the surface layer of MCM-41. In general, silicas can be functionalized in two ways: postsynthesis modification or direct co-condensation [27]. Among a large number of organic compounds for silica functionalization, cyclodextrin macromolecules are very promising because of their ability to form inclusion complexes with chemicals of suitable geometry and functionality [28].

Postsynthesis modification of silica surfaces was successfully realized by attachment of β-cyclodextrin or its derivatives to the silica support preliminary functionalized with *N*-(2-aminoethyl)-3-aminopropyl [29], carboxylated cuccinyl [30], 3-aminopropyl [31–35], 3-glycidoxypropyl [36, 37], hydrosilyl [38], ester [39], and 3*-*mercaptopropyl [40] groups, whereas the taken attempts to introduce β-cyclodextrin moieties into the silica framework by sol-gel methods involve the condensation of silica alkoxides with β-cyclodextrin [41] or β-cyclodextrin-containing silanes [42–44]. The predominant majority of these works relates to the synthesis of functionalized silica materials with disordered porous structure. However, usage of aggressive solvents and activating agents in multistep procedures of organic reactions at postsynthesis treatment as well as functional silanes and pore-expanding agents at sol-gel condensation process may affect the structure of final MCM-41-type materials substantially causing damage of their hexagonally ordered pore structure. At the same time, the chemical immobilization of β-cyclodextrin under mild conditions—through amide bond formation on macroporous silica—was carried out [45]. The activation properties of *N*,*N*′-carbonyldiimidazole in the reaction with β-cyclodextrin for the following immobilization of oligosaccharide derivative onto aminopropyl silica surface was used.

The idea of the present research was to use the possibility of β-cyclodextrin activation under mild conditions for preparing functionalized MCM-41-type silica materials with hexagonally ordered mesoporous structure. Here, two principal methods were exploited for β-cyclodextrin-containing MCM-41 silicas producing: postsynthesis attachment to the support by covalent bond formation or sol-gel synthesis using β-cyclodextrin-containing silane. First, aminopropyl-containing mesoporous ordered functionalized organosilica was prepared by co-condensation of tetraethyl orthosilicate and (3-aminopropyl)triethoxysilane, followed by postsynthesis grafting of β-cyclodextrin through the activating agent (*N*,*N*′-carbonyldiimidazole) usage. Another method of synthesis supposed the exploration of activated β-cyclodextrin for organosilane obtaining and its subsequent co-condensation with tetraethyl orthosilicate in the presence of cetyltrimethylammonium bromide to yield β-cyclodextrin-MCM-41 silica. Adsorption experiments were carried out to study the role of functionalization for effective uptake of benzene from water. It was expected that proposed synthetic approaches can be useful in preparing of MCM-41-type silica materials with high surface area, pore volume, and narrow pore size distribution as well as sufficient functional groups concentration.

## Methods

### Materials

β-cyclodextrin hydrate (β-CD) (99 %, Acros Organics), tetraethyl orthosilicate (TEOS) (≥99 %, Merck), (3-aminopropyl)triethoxysilane (APTES) (≥99 %, Merck), *N*,*N*′-carbonyldiimidazole (CDI) (≥98 %, Merck), and cetyltrimethylammonium bromide (CTMABr) (≥97 %, Merck) were used as purchased, and no further purification was performed. Aqueous ammonia (25 %), ethanol (96 %), and hydrochloric acid (37 %) were purchased from Reakhim and used without additional purification. Acetone (extra pure, Merck) and *N*,*N*′-dimethylformamide (DMF) (pure analytical, Reakhim) were dried for 48 h before utilization with activated molecular sieves (0.3 nm, Merck). Benzene (pure analytical, Reakhim) was used to prepare benzene solutions in water. Distilled water was used in all experiments.

### Postsynthesis Modification of MCM-41 Silica with β-CD Using Activation Agent

Hexagonally ordered NH_2_-MCM-41 silica support was prepared by hydrothermal sol-gel synthesis in the presence of ionic surfactant compound, CTMABr, by the procedure described in [15]. TEOS and APTES were used as silica sources. The final molar composition of the reaction mixture for NH_2_-MCM-41 silica preparing by template method was as follows: 0.09 TEOS:0.006 APTES:0.02 CTMABr:0.55 NH_4_OH:0.56 C_2_H_5_OH:14.4 H_2_O. Obtained amino-functionalized silica support was washed by water and dried at ambient temperature. Then, the template was removed by extraction in acid-ethanol solution. NH_2_-MCM-41 silica was dried in the air at 423 K for 4 h, cooled, and kept in a desiccator before use.

**Scheme 1:**
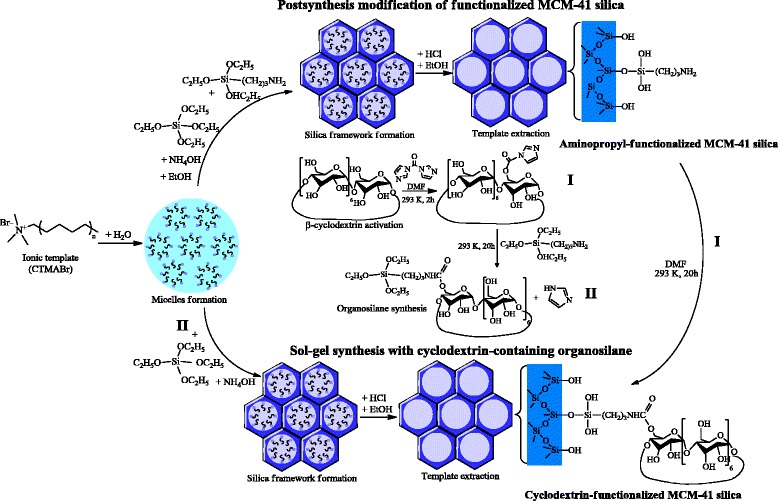
Synthesis of ordered β-cyclodextrin-containing MCM-41 silicas

Surface grafting of β-CD onto NH_2_-MCM-41 silica was carried out under mild conditions. First, β-CD was activated with CDI to form amide bonds [45]. To obtain activated oligosaccharide (**I**) (Scheme 1), a solution of CDI in dry DMF was added to a solution of anhydrous β-CD in dry DMF (molar ratio CDI:β-CD = 1:1) under continuous mixing. The activation reaction was carried out at 293 K for 2 h.

Then, reaction mixture with activated oligosaccharide (I) was slowly dropped into DMF suspension of NH_2_-MCM-41 silica with stirring. The grafting step lasted for 24 h at ambient temperature. Next, β-CD-grafted MCM-41 silica (CD-MCM-41_ps_) was filtered and washed sequentially with DMF, acetone, and distilled water. CD-MCM-41_ps_ was dried in the air at 293 K.

### Synthesis of CD-MCM-41 Silica Using β-CD Silane

The co-condensation method was also employed to incorporate β-CD in silica matrix. At the beginning, β-CD-containing organosilane (**II**) was prepared by modification of APTES with aforementioned activated oligosaccharide (**I**). Then, the obtained product was used for templated sol-gel synthesis of two types of CD-MCM-41_sg_ silicas. One of them, CD-MCM-41-1_sg_, was prepared by co-condensation of TEOS and **II** in the presence of activation reaction by-products. Meanwhile, the second silica (CD-MCM-41-2_sg_) was synthesized by use of TEOS and purified β-CD-organosilane. Purification of β-CD-organosilane was realized in accordance with the following procedure. Dry acetone was added to the reaction mixture of β-CD-containing organosilane (**II**) to fall out substituted oligosaccharide [44, 46], and the resultant precipitate was collected by filtration. After drying under vacuum, the yellow solid product was obtained. Finally, purified β-CD-organosilane was dissolved in dry DMF and co-condensed with TEOS in the presence of CTMABr. Ionic template was previously dissolved in water with stirring at room temperature, and NH_4_OH was added to provide the alkaline medium of the reaction.

The reaction mixtures for both CD-MCM-41-1_sg_ and CD-MCM-41-2_sg_ silicas were agitated on magnetic stirrer for 2 h. In order to complete the condensation process, the hydrothermal treatment in autoclave at 373 K for 24 h was carried out. The final molar composition of the reaction mixture for CD-MCM-41_sg_ silicas preparing was as follows: 0.05 TEOS:0.001 β-CD-organosilane:0.007 CTMABr:0.27 NH_4_OH:7.2 H_2_O. Both CD-MCM-41_sg_ materials were washed by small quantities of water and dried at ambient temperature. Then, the template was removed by triple solvent extraction in HCl/C_2_H_5_OH solution at room temperature for 24 h. After extraction, silicas were washed with distilled water until the negative test for halogenide anions with AgNO_3_. Obtained materials were dried in the air at 293 K.

### Characterization

The ordered mesoporosity of the aminopropyl- and β-CD-containing silicas was confirmed by diffraction analysis at low angles (2*θ* = 1–10 grad) and transmission electron microscopy (TEM).

Powder X-ray diffraction patterns (XRD) were measured on a DRON-4-02 diffractometer using CuKα radiation (*λ* = 0.154178 nm) and a nickel filter.

TEM experiments were carried out on a JEM JEOL 1230 electron microscope operated at 100 kV. The samples (0.05 g) for TEM measurements were suspended in ethanol (4 ml) and processed with ultrasonic treatment for 3 min (ultrasound power 60 W). Obtained suspensions (50 μL) were supported onto formvar film on a Cu grid, followed by drying at ambient conditions.

The transmission spectra were registered on a Thermo Nicollet NEXUS Fourier transform infrared (FT-IR) spectrophotometer in the range from 4000 to 400 cm^–1^ for solid pellets of MCM-41-type silicas.

Porosity measurements were obtained with a Kelvin-1042 Sorptometer using low-temperature adsorption-desorption of nitrogen. Prior to measurements, all samples were outgases at 413 K for 20 h.

Specific surface area of CD-MCM-41 silicas was determined using the BET method in the relative pressure range (*P*/*P*_0_) up to 0.30. The pore size distributions were calculated by applying the non-localized density functional theory (NLDFT) (equilibrium model). The total pore volume (*V*_total_) was obtained from the amount of nitrogen adsorbed at *P*/*P*_0_ = 0.99.

The amount of surface aminopropyl groups was calculated by the difference in pH values (ionometer I-160) of starting and equilibrium acid solutions with CD-MCM-41_ps_ (or CD-MCM-41_sg_) silica batch after 24-h contact [47].

The content of β-CD groups chemically immobilized on the surface of CD-MCM-41 silicas was defined by acid hydrolysis of cyclodextrin up to glucose. The concentration of glucose after the reaction with potassium ferrocyanide was defined by spectrophotometry using Specord M-40 equipment (Germany, Carl Zeiss, Jena) at *λ* = 420 nm [45, 48].

### Adsorption Studies

The treatment of a dilute solution of benzene (0.38 g · L^–1^) with synthesized MCM-41-type silica materials was investigated. The adsorption behavior of benzene was studied on pristine MCM-41 silica, amino-functionalized silica support NH_2_-MCM-41, and ordered β-CD-containing silicas (CD-MCM-41_ps_, CD-MCM-41-1_sg_, and CD-MCM-41-2_sg_). For each silica composition, 0.035 g of sample was stirred with 25 ml of benzene aqueous solution for 24 h at 291 K in air-tight glass vials. Then, the suspensions were filtered through syringe filters (pores with *d* = 0.2 μm, PVDF (Millipore)) to prevent the liberation of aromatic compound, and the quantities of benzene in filtrates were determined by UV-spectrophotometry at *λ* = 254 nm. The amount of benzene adsorbed on MCM-41-type silicas was evaluated as:

$$ a=\frac{\left({C}_0-{C}_f\right)V}{m}, $$

where *a* is the adsorption, *C*_*0*_ is the initial concentration of benzene, *C*_*f*_ is the concentration of benzene in filtrate, *V* is the volume of the aqueous solution of benzene, and *m* is the mass of adsorbent.

The kinetic and adsorption equilibrium studies for MCM-41, NH_2_-MCM-41, CD-MCM-41-1_sg_, and CD-MCM-41-2_sg_ silicas were realized by the multibatch method at 291 K. For kinetic experiments, air-dried weighted amounts (0.02 g) of each silica were taken in air-tight vials; then, 12 ml of benzene aqueous solution (0.45 g · L^–1^) was rapidly added. The suspensions were stirred for predetermined time intervals, and the filtrates were analyzed spectrophotometrically. The concentration of benzene in filtrate solution was calculated from the calibration curve prepared by plotting absorbance at 254 nm of various known concentration of benzene aqueous solutions (0.01–0.45 g · L^–1^). For equilibrium adsorption experiments, aqueous solution of benzene with concentration in the range 0.036–0.72 g · L^–1^ were used. Briefly, 0.01 g of air-dried silica adsorbent was taken in air-tight vials and 12 ml of aqueous solution of benzene was added to it. After the absorption equilibrium was reached, the solution was extracted by syringe filter for determination of benzene concentration. The standard calibration curves were used to calculate equilibrium concentration of benzene solutions from UV absorbance intensity at *λ* = 254 nm. The experimental errors of benzene concentrations in the solutions were calculated as follows:

$$ \frac{\varDelta C}{C}=\frac{\varDelta A+\varDelta B\cdot D+B\cdot \varDelta D}{D}, $$

where *ΔС* is the absolute error; *А*, *В* and Δ*А*, Δ*В* are the coefficients obtained from the calibration curve (an intercept and the slope) and their absolute errors at confident level 0.95, correspondingly; and Δ*D* was accepted as 0.005 (device error). Values of benzene adsorption on the surface of silica adsorbents were calculated as described previously.

## Results and Discussion

**Fig. 1:**
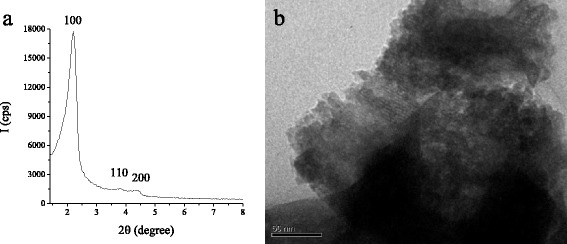
XRD pattern (**a**) and TEM image of NH_2_-MCM-41 silica (**b**)

Ordered mesoporous silicas are commonly used for the production of functionalized materials, owing to their unique properties including a regular mesostructure along with high specific surface areas, thermal and mechanical stability, highly uniform pore distribution and tunable pore size, as well as high adsorption capacity. In this work, the syntheses of functional β-CD-containing MCM-41 silicas were attempted to combine the forenamed properties of ordered material and macromolecule peculiar to form inclusion complexes with a wide variety of organic/inorganic compounds. The new β-CD-containing MCM-41 silicas with hexagonally ordered porous structure were prepared using activation properties of CDI by two different methods: postsynthesis modification of functionalized MCM-41 and direct co-condensation of β-CD-containing organosilane and TEOS (Scheme 1). Grafting of organic macromolecule β-CD was carried out on the surface of preconstituted mesoporous MCM-41 silica with covalently bonded amino groups. Sol-gel hydrothermal synthesis in the presence of a structure directing agent was chosen for NH_2_-MCM-41 support obtaining for better control of the loading of amino moieties by desired TEOS/APTES ratio selection. The ordered porosity of NH_2_-MCM-41 support was confirmed by XRD and TEM analyses (Fig. 1). The XRD pattern of NH_2_-MCM-41 support after template extraction (Fig. 1a) exhibits an intense signal at 2*θ* = 2.2 grad. corresponding to (100) plane and two small signals between 3.5 and 5 grad. due to (110) and (200) planes which prove the presence of well-defined hexagonal structure of MCM-41 [1]. Also, the array of tunable pores could be seen in NH_2_-MCM-41 silica microphotography (Fig. 1b). The quantity of the aminopropyl groups introduced into the silica surface equals 0.44 mmol · g^−1^.

Chemical bonding of β-CD with NH_2_-MCM-41 silica support was realized with coupling agent participation. Since hydroxyl groups of β-CD exhibit great reactivity, the formation of C(O)–N bonds under the influence of CDI activator occurred even at ambient temperature (Scheme 1). The presence of imidazole by-product in the reaction mixture during the grafting on silica support does not lead to drastical changes in pH, and NH_2_-MCM-41 silica structure did not alter. Imidazole was easily removed from the surface of CD-MCM-41_ps_ silica by washing with organic solvents and distilled water.

Activated β-CD was also used for obtaining the proper organosilane for subsequent co-condensation with TEOS in attendance of CTMABr template. To confirm that by-products of β-CD activation reaction could not affect the structure of the final silica, two types of CD-MCM-41 silicas were prepared by sol-gel synthesis. The nature of surface layer and mesoporous structure of CD-MCM-41-1_sg_ and CD-MCM-41-2_sg_ silicas obtained from β-CD-alkoxide with by-products and purified one, correspondingly, were compared.

**Fig. 2:**
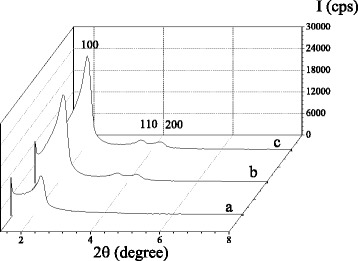
XRD patterns of CD-MCM-41_ps_ (*a*), CD-MCM-41-1_sg_ (*b*), and CD-MCM-41-2_sg_ (*c*) silicas

Characterization of CD-MCM-41_ps_, CD-MCM-41-1_sg_, and CD-MCM-41-2_sg_ prepared by two methods was performed using XRD, TEM, FT-IR spectroscopy, chemical analysis, and low-temperature adsorption-desorption of nitrogen. Hexagonally ordered pore structure of obtained silicas was confirmed by XRD analysis. XRD patterns of synthesized CD-MCM-41 silicas are shown in Fig. 2. The presence of diffraction peaks at 2*θ* = 2.20, 2.15, and 2.15 grad. are attributed to the (100) reticular planes in CD-MCM-41_ps_, CD-MCM-41-1_sg_, and CD-MCM-41-2_sg_ silicas, respectively. Moreover, distinct reflexes on the XRD patterns of CD-MCM-41_sg_ between 3.5 and 5 grad. could be indexed to the (110) and (200) reticular planes of hexagonally packed pores and confirm the formation of two-dimensionally periodic hexagonal lattice, which is characteristic for MCM-41. The most intensive (100) reflex for CD-MCM-41_ps_ is slightly shifted to high-angle region compared with CD-MCM-41_sg_ materials, evidencing the reduction of interplanar distances *d* in silica framework. Structural parameters (interplanar distance *d* and unit cell parameter *a*) calculated from XRD analysis for all silicas are summarized in Table 1. Even if the diffraction peaks positions are similar in XRD patterns of CD-MCM-41_ps_ and CD-MCM-41_sg_ silicas, the difference in their intensity indicates a lower mesoporous structure ordering of CD-MCM-41_ps_. Thus, co-condensation method gives β-CD-functionalized MCM-41 with higher long-range order of the hexagonal pore arrays. Furthermore, the same values of *d* and *a* diffraction parameters for CD-MCM-41_sg_-1 and CD-MCM-41_sg_-2 silicas denote that the presence of by-products in β-CD-containing alkoxide mixture involved into the sol-gel synthesis has no noticeable effect on the formation of ordered hexagonal network of pores.

**Table 1:** Structural properties of ordered β-cyclodextrin-containing MCM-41 silicas

Silica	*d*_100_ (nm)	*a* (nm)	*S*_BET_ (m^2^ · g^−1^)	*V*_total_ (cm^3^ · g^−1^)	*D*_DFT_ (nm)		[–(CH_2_)_3_NH_2_]		[β-CD]	
							(mmol · g^−1^)	(μmol · m^−2^)	(mmol · g^−1^)	(μmol · m^−2^)
CD-MCM-41_ps_	4.02	4.64	580	0.97	3.4	5.1	0.43	0.74	0.016	0.028
CD-MCM-41-1_sg_	4.11	4.75	812	1.06	3.9	5.1	0.05	0.06	0.018	0.022
CD-MCM-41-2_sg_	4.11	4.75	802	1.38	3.9	5.2	0.04	0.05	0.010	0.012

**Fig. 3:**
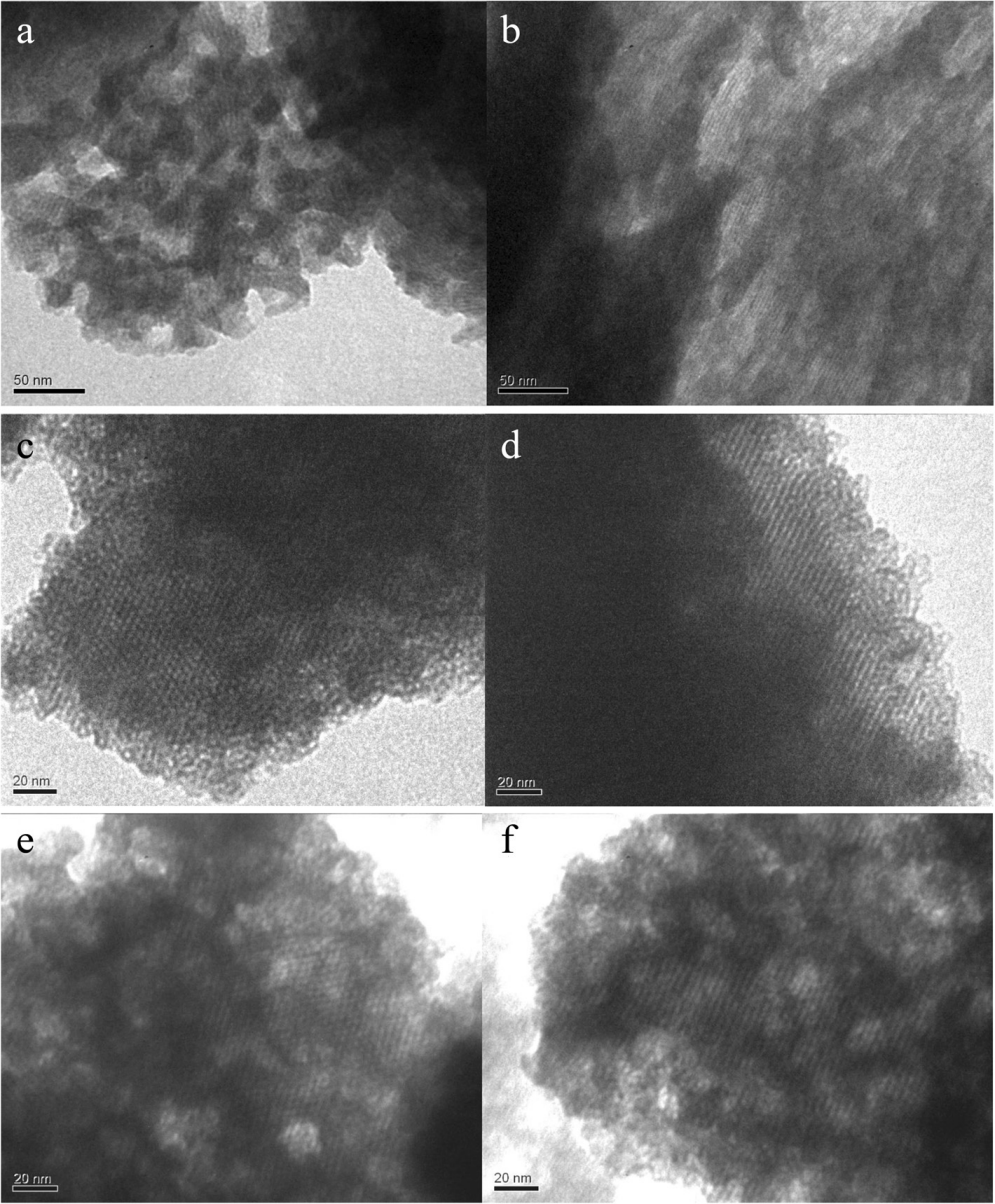
TEM images of CD-MCM-41_ps_ (**a**, **b**), CD-MCM-41-1_sg_ (**c**, **d**), and CD-MCM-41-2_sg_ (**e**, **f**) silicas

The XRD results were also verified by TEM analysis. The lower ordering degree was seen on the TEM images of CD-MCM-41_ps_ silica in comparison with CD-MCM-41_sg_ ones (Fig. 3a,b). As could be clearly seen from Fig. 3c,e, the unidimensional cylindrical pores of CD-MCM-41_sg_ silicas are arranged in a honeycomb structure. On the TEM images taken perpendicular to the pore channels (Fig. 3d,f), the long-range array of tubular voids is observed. Synthesized hexagonally ordered β-CD-containing MCM-41 silicas possess sheet-like morphology.

**Fig. 4:**
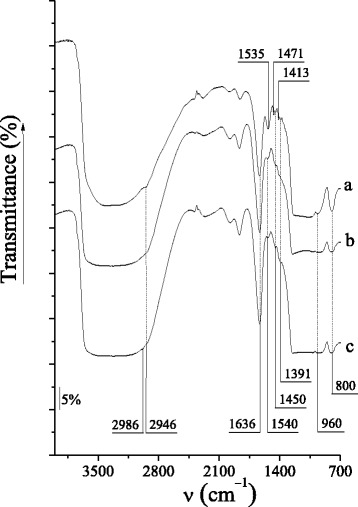
IR spectra of CD-MCM-41_ps_ (*a*), CD-MCM-41-1_sg_ (*b*), and CD-MCM-41-2_sg_ (*c*) silicas

Chemical immobilization of cyclic oligosaccharide groups on the surface of silica materials of MCM-41-type was proved by FT-IR spectroscopy. In the FT-IR spectra of CD-MCM-41_ps_ and CD-MCM-41_sg_ silicas (Fig. 4), the absorption bands at 2986, 2946 and 1471, 1450, 1413, 1391 cm^–1^ corresponding to the valence and deformation vibrations of the C–H bonds in the alkyl and glycosyl groups of grafted compounds are registered. Moreover, the characteristic absorption bands at 1535 and 1540 cm^−1^ belonging to the deformation vibrations of the N–H bond in the secondary amino groups of CD-MCM-41_ps_ and CD-MCM-41_sg_ silicas, respectively, are clearly shown. Obviously, their presence is caused by the chemical immobilization of β-CD-containing groups in the surface layer of silica support. Absorption bands attributed to the deformation vibrations of the N–H bond in the residual primary amino groups (1560–1640 cm^−1^) as well as the valence vibrations of the C=O bond in the amide linkage (nearly by 1700 cm^−1^) [49] of synthesized silicas are not registered because of their overlapping with strong signal attributed to the deformation vibration of the O–H bond in the adsorbed water molecules. The silanol groups disposed on the silica surface and remaining water molecules produce the broad stretching band around 3000–3600 cm^–1^, followed by bands at 1636 and 960 cm^−1^, attributed to the deformation vibrations of the O–H bonds.

The evidence of MCM-41 silicas functionalization by two proposed synthesis methods was also demonstrated due to chemical analysis of surface compounds. The estimated content of aminopropyl and β-cyclodextrin groups on the surface of synthesized materials is summarized in Table 1. It can be seen that only a small part of aminopropyl groups for CD-MCM-41_ps_ has been reacted with activated β-CD. So many unreacted amino groups for CD-MCM-41_ps_ may indicate that β-CD is mainly grafted at the entrance of the pores, preventing further penetration of oligosaccharide moieties to the inner pore surface with other anchoring sites [42]. The appearance of aminopropyl groups in CD-MCM-41_sg_ (Table 1) points to the partial hydrolysis of amide bonds under hydrothermal treatment of β-CD-containing silicas in the medium of ammonium. It is evident that postsynthesis grafting as well as sol-gel synthesis leads to bifunctional MCM-41 silica obtaining.

**Fig. 5:**
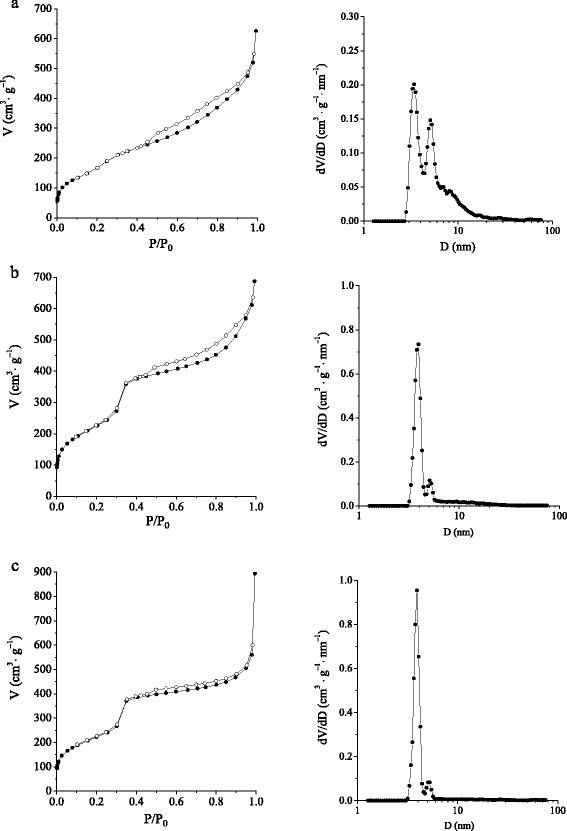
Nitrogen adsorption-desorption isotherms and NLDFT pore diameter (on the *right*) of CD-MCM-41_ps_ (**a**), CD-MCM-41-1_sg_ (**b**), and CD-MCM-41-2_sg_ (**c**) silicas. *Solid symbols* denoted adsorption, and *open symbols* denoted desorption

Low-temperature adsorption-desorption of nitrogen was used to investigate the pore structure of CD-MCM-41 silicas. The isotherms of nitrogen adsorption-desorption as well as the pore size distributions of synthesized silicas are shown in Fig. 5. As can be seen from the isotherm of CD-MCM-41_ps_ silica, a gradual rise of nitrogen adsorption at low relative pressures (up to 0.4) with slight visible inflection step takes place. It is accounted for the sequential formation of adsorbate monolayers on the walls of mesoporous channels with different sizes. The pore size distribution plot calculated by the NLDFT model confirms the complex pore structure of grafted silica. For CD-MCM-41_ps_ silica, the broad pore size distribution with two distinct (at 3.4 and 5.1 nm) peaks is observed suggesting the existence of two types of mesopores as well as textural porosity within the sheet-like particles (Fig. 5a). Nonetheless, CD-MCM-41_ps_ has a high surface area (580 m^2^ · g^−1^) and pore volume (0.97 cm^3^ · g^−1^). Figure 5b, c displays isotherms of nitrogen adsorption-desorption and pore size distributions for CD-MCM-41-1_sg_ and CD-MCM-41-2_sg_ silicas. Nitrogen adsorption at low relative pressures *P*/*P*_0_ < 0.3 for CD-MCM-41-1_sg_ silicas is attributed to the monolayer adsorption, following multilayer adsorption on the walls of mesopores. The distinct step on the isotherm at *P*/*P*_0_ ~ 0.35 indicates a uniformly porous surface. The pore size distribution plots clearly demonstrate that uniform pores are prevailing in CD-MCM-41_sg_ materials causing the high peak centered at 3.9 nm. The appearance of larger pores (slight peak above 5 nm) can be explained by partial degradation of the walls between individual channels of pores in the process of postsynthesis treatment carried out at 373 K in the medium of ammonia. Also, the kind of isotherms of nitrogen adsorption-desorption for CD-MCM-41-1_sg_ silica confirms that by-products of β-CD activation reaction do not affect the pore structure of the resulting silica. The values of BET specific surface area for CD-MCM-41_sg_ silicas calculated from linear region of isotherms are more than 800 m^2^ · g^−1^. It is worth noting that β-CD-containing silicas with high surface areas, pore volume, and large-scale ordering was managed to get by sol-gel method without any pore-expanding agent. Finally, it could be concluded that grafted CD-MCM-41_ps_ is generally less ordered than CD-MCM-41-1_sg_ silica with the similar concentration of β-CD groups.

The existence of functional groups within the framework of MCM-41 materials causes the change in the surface chemistry and porosity of the solid, which in turn affects the sorption behavior of MCM-41-type silicas. Earlier [50], we used UV spectroscopy to prove that β-CD and benzene could form 1:1 “host–guest” inclusion complex in aqueous solution. It was shown that formation of “β-CD-benzene” complex is spontaneous and thermodynamically profitable exothermal process. Incorporation of cyclic oligosaccharide in solid supports like MCM-41 silicas makes possible an efficient removing of aromatic pollutants from aqueous solutions by means of supramolecular structure formation.

**Fig. 6:**
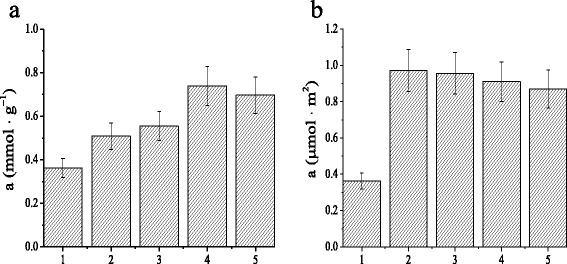
Adsorption effectiveness of MCM-41 (*1*), NH_2_-MCM-41 (*2*), CD-MCM-41_ps_ (*3*), CD-MCM-41-1_sg_ (*4*), and CD-MCM-41-2_sg_ (*5*) silicas in dilute benzene solution (0.38 g · L^−1^) per gram (**a**) or per square meter (**b**) of materials

Here, adsorption behaviors of functionalized MCM-41 silicas were studied by benzene withdrawal from aqueous solutions. Table 2 shows the adsorption capacity of pristine MCM-41 and functionalized MCM-41 silicas. It was demonstrated that the uptake of benzene molecules is caused by non-specific binding interaction with silica matrix as well as selective binding sites within its structure. The adsorption of benzene on silicas based on interactions of the aromatic ring of π-electrons with the surface silanol groups is well known [51]. Simultaneously, the increase in adsorption capacity of NH_2_-MCM-41 silica toward benzene molecules could be explained by hydrophobic interaction between aromatic molecules and carbon chains of aminopropyl fragments. It was found out that introduction of cyclodextrin moieties in silica structure leads to the increase of benzene adsorption (Fig. 6a). However, taking into account the difference in surface areas of silicas, it is difficult to elucidate the contribution of surface cyclic oligosaccharide groups to the uptake of aromatic compounds (Fig. 6b). Obviously, a large number of unreacted amino groups in CD-MCM-41_ps_ prevent to evaluate the evidence that adsorption of benzene is driven by noncovalent interaction of guest molecules with attached to silica matrix oligosaccharide host. Therefore, adsorption kinetics and equilibrium adsorption isotherms were investigated for non-modified MCM-41, aminopropyl functionalized NH_2_-MCM-41, and two types of cyclodextrin-containing CD-MCM-41_sg_ silicas.

**Table 2:** Adsorption properties of ordered MCM-41 silicas in dilute benzene solution (0.38 g · L^−1^)

Silica	*S*_BET_ (m^2^ · g^−1^)	*a* (mmol · g^−1^)	*a* (μmol · m^−2^)
MCM-41	995	0.361 ± 0.043	0.363 ± 0.117
NH_2_-MCM-41	523	0.508 ± 0.061	0.972 ± 0.117
CD-MCM-41_ps_	580	0.555 ± 0.061	0.956 ± 0.115
CD-MCM-41-1_sg_	812	0.739 ± 0.089	0.910 ± 0.109
CD-MCM-41-2_sg_	802	0.696 ± 0.084	0.870 ± 0.104

**Fig. 7:**
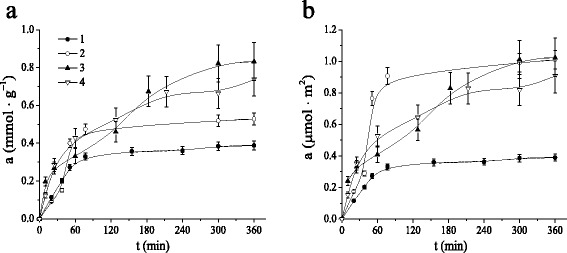
Kinetic curves of benzene adsorption on MCM-41 (*1*), NH_2_-MCM-41 (*2*), CD-MCM-41-1_sg_ (*3*), and CD-MCM-41-2_sg_ (*4*) silicas per gram (**a**) or per square meter (**b**) of materials

Adsorption kinetic experiments were carried out to evaluate a period which should be sufficient to reach adsorption equilibrium. Figure 7 represents the kinetic curves for benzene uptake from aqueous solutions with MCM-41, NH_2_-MCM-41, CD-MCM-41-1_sg_ and CD-MCM-41-2_sg_ silicas. The adsorption of benzene on MCM-41 and NH_2_-MCM-41 silicas is characterized by rapid uptake of aromatic molecules within the first hour until it slows down and become constant at 5 h. For CD-MCM-41_sg_ silicas, adsorptive uptake increases slowly and becomes constant in about 6 h.

**Fig. 8:**
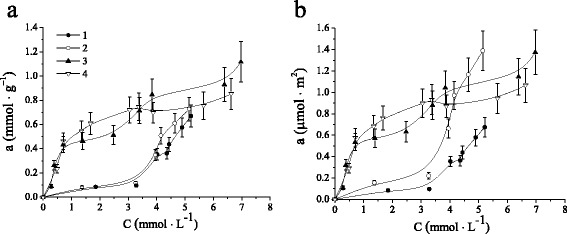
Benzene adsorption isotherms on MCM-41 (*1*), NH_2_-MCM-41 (*2*), CD-MCM-41-1_sg_ (*3*), and CD-MCM-41-2_sg_ (*4*) silicas per gram (**a**) or per square meter (**b**) of materials

The equilibrium relationships between MCM-41 adsorbents and benzene were described by sorption isotherms. The adsorption isotherms of benzene from aqueous solutions as quantity of benzene adsorbed per unit weight of silica or as quantity of benzene adsorbed per unit of adsorbent surface are given in Fig. 8a and in Fig. 8b, respectively. It can be seen that adsorption isotherms of MCM-41 and NH_2_-MCM-41 silicas have concave shape in the region of small equilibrium concentrations of benzene as a result of weak affinity of non-polar aromatic ring to polar silanol groups, but then followed a sharp increase of aromatic uptake. Obviously, it could be explained by the reorientation of benzene molecules owing to increase its concentration in solution, and the more benzene is already adsorbed, the easier it is for additional amounts to become fixed as a result of hydrophobic interaction [52].

The character of benzene adsorption on the surface of CD-MCM-41_sg_ silicas is considerably different. In the region of small equilibrium concentrations, the higher adsorption of benzene compared to MCM-41 and NH_2_-MCM-41 silicas is observed. This signifies that CD-MCM-41_sg_ silicas can act as effective adsorbents for low levels of aromatic molecules. The equilibrium adsorption isotherms of benzene on CD-MCM-41-1_sg_ and CD-MCM-41-2_sg_ silicas were analyzed by use of Langmuir and Freundlich models of adsorption. A linear form of the Langmuir and Freundlich equations, respectively, were used to determine isotherm parameters:

$$ \frac{C_{\mathrm{eq}}}{a_{\mathrm{eq}}}=\frac{1}{a_m{K}_L}+\frac{C_{\mathrm{eq}}}{a_m}, $$

$$ \ln {a}_{\mathrm{eq}}= \ln {K}_F+\frac{1}{n} \ln {C}_{\mathrm{eq}}, $$

where *C*_eq_ is the equilibrium concentration of adsorptive in a solution (mg · L^−1^), *a*_eq_ is the equilibrium adsorption (mg · g^–1^), *K*_*L*_ is the Langmuir constant that characterizes the adsorption energy (L^−1^ · mg), *a*_*m*_ is the adsorption capacity of monolayer (mg · g^−1^), *K*_*F*_ is the Freundlich constant (L^−1^ · mg), and 1/n is the Freundlich constant characteristic of adsorption intensity. The calculated parameters from both models are summarized in Table 3. As expected, CD-MCM-41-1_sg_ has the higher adsorption capacity toward benzene.

**Table 3:** Parameters of benzene adsorption calculated by Langmuir and Freundlich equations for ordered β-cyclodextrin-containing MCM-41 silicas

Silica	Langmuir model			Freundlich model		
	*K*_L_ (L · mg^−1^)	*a*_m_ (mg · g^−1^)	*R* ^2^	*K*_F_ (L · mg^−1^)	*n*	*R* ^2^
CD-MCM-41-1_sg_	0.019	111	0.83	1.9	1.64	0.84
CD-MCM-41-2_sg_	0.015	74	0.98	6.0	2.53	0.80

It was shown that typical adsorption capacities for activated carbon and silica adsorbent in liquid phase under different conditions are in the range of 12–230 mg · g^−1^ for benzene [53, 54]. Therefore, prepared cyclodextrin-containing MCM-41 silicas demonstrate adsorption level performance of known samples and could be very promising for the treatment of aqueous solutions with low benzene concentration.

## Conclusions

In this research, we realized two principal methods of β-cyclodextrin-functionalized MCM-41-type silicas producing: postsynthesis attachment to the support by covalent bond formation or sol-gel synthesis using β-cyclodextrin-containing silane in the presence of ionic template. β-Cyclodextrin activated by *N*,*N*′-carbonyldiimidazole was employed for both synthetic approaches. Obtained functional materials were characterized by XRD, TEM, and chemical analyses, FT-IR spectroscopy, and low-temperature adsorption-desorption of nitrogen. The results of this study indicate that co-condensation method leads to the formation of MCM-41 silicas with higher arrangement of mesoporous channels compared with one obtained by postsynthesis grafting. Moreover, it was proved that by-products of β-CD activation reaction could not affect the structure of the final silica. The proposed synthesis approaches may be applicable for obtaining of ordered β-cyclodextrin-containing functional materials with high affinity to chemicals of suitable geometry. Adsorption study of benzene uptake from aqueous solutions confirms the probability of β-cyclodextrin-functionalized MCM-41-type silica use in water treatment processes.
